# LRRK2 Expression Is Deregulated in Fibroblasts and Neurons from Parkinson Patients with Mutations in PINK1

**DOI:** 10.1007/s12035-016-0303-7

**Published:** 2016-12-14

**Authors:** Garikoitz Azkona, Rakel López de Maturana, Patricia del Rio, Amaya Sousa, Nerea Vazquez, Amaia Zubiarrain, Daniel Jimenez-Blasco, Juan P. Bolaños, Blas Morales, Georg Auburger, José Matias Arbelo, Rosario Sánchez-Pernaute

**Affiliations:** 1 Laboratory of Stem Cells and Neural Repair, Inbiomed, Paseo Mikeletegi, 81, 20009 San Sebastian, Spain; 2 Animal Model Unit, Inbiomed, San Sebastian, Spain; 3 Present Address: Animal Research Facility, Scientific and Technological Centers, University of Barcelona, Barcelona, Spain; 4 Institute of Functional Biology and Genomics (IBFG), University of Salamanca—CSIC, Salamanca, Spain; 5 Institute of Biomedical Research of Salamanca (IBSAL), University Hospital of Salamanca, Salamanca, Spain; 6 Department of Neurology, University Hospital San Cecilio, Granada, Spain; 7 Experimental Neurology, Goethe University Medical School, Frankfurt am Main, Germany; 8 Parkinson’s and Movement Disorders Unit, Department of Neurology, Hospital Universitario Insular de Gran Canaria, Las Palmas de Gran Canaria, Spain; 9 Present Address: Andalusian Initiative for Advanced Therapies, Junta de Andalucia, Sevilla, Spain

**Keywords:** Parkinson disease, iPSC, PINK1, LRRK2

## Abstract

Mutations in *PINK1* (*PARK6*), a serine/threonine kinase involved in mitochondrial homeostasis, are associated with early onset Parkinson’s disease. Fibroblasts from Parkinson’s disease patients with compound heterozygous mutations in exon 7 (c.1488 + 1G > A; c.1252_1488del) showed no apparent signs of mitochondrial impairment. To elucidate changes primarily caused by lack of functional PINK1, we over-expressed wild-type *PINK1*, which induced a significant downregulation of *LRRK2* (*PARK8*). Indeed, we found that LRRK2 protein basal levels were significantly higher in the mutant *PINK1* fibroblasts. To examine the interaction between the two *PARK* genes in a disease-relevant cell context, we generated induced pluripotent stem cell (iPSC) lines from mutant, carrier and control fibroblasts by lentiviral-mediated re-programming. Efficiency of neural induction and dopamine differentiation using a floor-plate induction protocol was similar in all genotypes. As observed in fibroblasts, *PINK1* mutant neurons showed increased LRRK2 expression both at the RNA and protein level and transient over-expression of wild-type *PINK1* efficiently downregulated *LRRK2* levels. Additionally, we confirmed a dysregulation of *LRRK2* expression in fibroblasts from patients with a different homozygous mutation in *PINK1* exon 4, c.926G > A (G309D). Thus, our results identify a novel role of PINK1 modulating the levels of LRRK2 in Parkinson’s disease fibroblasts and neurons, suggest a convergent pathway for these *PARK* genes, and broaden the role of LRRK2 in the pathogenesis of Parkinson’s disease.

**Electronic supplementary material:**

The online version of this article (doi:10.1007/s12035-016-0303-7) contains supplementary material, which is available to authorized users.

## Introduction

Rare monogenic forms of Parkinson’s disease (PD) have been correlated to specific gene mutations [1], providing the opportunity to identify novel pathogenic pathways or molecular mechanisms that may contribute to more frequent forms of the disease [2] and to examine interactions between *PARK* genes. Alternatively, early-onset genetic forms could represent a distinct entity at the molecular level.

PD-related mutations in *PINK1* (PTEN-induced kinase 1, *PARK6*) compromise kinase function or protein stability [3]; thus, the phenotype is thought to result from a loss of function. PINK1 mitochondrial localization supports its involvement in the mitochondrial dysfunction extensively described in PD [4], and PINK1 protects against stress-induced mitochondrial dysfunction [3, 5]. In addition, the PINK1/Parkin signaling pathway controls mitochondrial quality and mitophagy [6], even if it appears that this pathway is not modified by endogenous human *PINK1* mutations [7, 8]. Likewise, PINK1 regulates mitochondrial fusion and fission dynamics [9], although the net effect of PINK1 mutations is currently a matter of controversy because both increased fusion and increased fission have been reported in different species [10].

*LRRK2* (*PARK8*) is one of the genes most frequently mutated in PD. LRRK2 has been implicated in a very broad range of cellular pathways so the precise mechanisms leading to neuronal degeneration remain to be defined [11]. An effect of LRRK2 on mitochondrial function was described in *Caenorhabditis* *elegans* [12]. Interestingly, in this model organism, the absence of lrk-1 rescues all phenotypic aspects of pink-1 loss-of-function mutants. Conversely, the hypersensitivity of lrk-1 mutant animals to stressors is reduced in a pink-1 mutant background, suggesting antagonistic effects of lrk-1 and pink-1 [13].

With the aim to define a PINK1-related phenotype for in vitro studies, we investigated expression pattern of genes involved in mitochondrial dynamics and *PARK* genes in fibroblasts and induced pluripotent stem cell (iPSC)-derived dopamine neurons from a PINK1-PD Spanish kindred [14]. This led us to unveil an upregulation of LRRK2 in *PINK1* mutants and an interaction between these two *PARK* gene products in human cells which were ratified in a different pedigree.

## Methods

### Human Samples

Skin samples were obtained from subjects expressing mutated forms of *PINK1* diagnosed at the Hospital Universitario Insular de Gran Canaria (La Palma de Gran Canaria, Spain) and from age-matched healthy individuals at the Hospital Donostia and Onkologikoa (San Sebastian, Spain). PD patients presented an early-onset, typical parkinsonian syndrome, characteristic of PINK1-associated PD [14]. Demographic data are provided in Table 1. Three samples from individuals from another Spanish family harboring the G309D (c.926G > A) mutation in exon 4 [3] were also analyzed to avoid possible confounding effects caused by consanguinity in the first family. Dermal fibroblasts were cultivated as described previously [15].

**Table 1:** Dermal fibroblast samples

Human fibroblast samples	Age at biopsy	Pathology
M-35	<55	None
M-44	<55	None
FH1103	<55	None
FH0819	>55	None
FH0821	>55	None
PDP1: PINK1^c.1488 + 1G > A + c.1252_1488^ (PINK1^-exon7/del^)	<55	PD
PDP2: PINK1^c.1488 + 1G > A^ (PINK1^-exon7^)	<55	Asymptomatic
PDP3: PINK1^c.1488 + 1G > A + c.1252_1488^ (PINK1^-exon7/del^)	>55	PD
PDP4: PINK1^c.1488 + 1G > A^ (PINK1^-exon7^)	<55	Asymptomatic
PDP5: PINK1 G309D/G309D	>55	PD
PDP6: PINK1 G309D	>55	Asymptomatic
PDP7: PINK1 G309D/G309D	<55	PD

The study included dermal fibroblasts from five control subjects (two males and three females) and seven subjects with mutations in PINK1 from two Spanish kindreds, four patients (two males and two females and three carriers (one male and two females) [14, 20]. Fibroblasts from the PDP1, PDP2, and PDP3 individuals were reprogrammed to obtain iPSCs lines and subsequently differentiated to dopamine neurons

*PD* Parkinson disease

### Protocol Approvals, Registrations, and Patient Consents

The study was approved by the Ethical Committee on the Use of Human Subjects in Research in Euskadi, Spain. All subjects gave informed consent for the study using forms approved by the ethical committees on the Use of Human Subjects in Research at Hospital Universitario Insular de Gran Canaria, La Palmas de Gran Canaria; Hospital Donostia and Onkologikoa, San Sebastián; and Hospital San Cecilio, Granada, respectively. Generation of iPSC lines was approved by the *Advisory Committee for Human Tissue and Cell Donation and Use*, Instituto Carlos III, Ministry of Health, Spain.

### Genetic Analysis

*PINK1* variants were analyzed by conventional PCR using a primer pair designed to amplify a region expanding exons 6 and 8 [14]. Total RNA and cDNA were obtained as described previously [15] for quantitative RT-PCR. Primer sequences [14] are provided in Supplementary Table S1. Comparative analysis of gene expression levels (∆∆Ct) was carried out using GAPDH as reference. Standard G-band karyotypes of the iPSC clones used in the study were performed at the Policlinica Gipuzkoa (San Sebastian, Spain).

### ATP Content

Cellular ATP was measured using the Luminiscent ATP Detection kit (Abcam, Cambridge, UK). Cells were harvested, pelleted, and washed once in PBS. An aliquot was used for protein quantification. The rest was re-suspended in 50 μl growth medium, and cells were lysed by adding 50 μl of the detergent and mixing. After a 5-min incubation, 50 μl of substrate was added and luminescence was quantified in a GloMax® luminometer (Promega, Madison, WI, USA). An ATP standard curve was prepared, and values were calculated in picomole per microgram of protein in the cell extract.

### Glycolytic Rate

The rate of glycolysis was determined in fibroblasts, seeded in flasks, by the conversion of [3-^3^H] glucose into ^3^H_2_O, as described previously [16].

### Western Blotting

Whole-cell lysates were prepared in RIPA buffer with a protease inhibitor cocktail (Roche, Mannheim, Germany). SDS-PAGE and protein transfer and blotting were carried out according to standard procedures [15]. Primary and secondary antibodies are listed in Supplementary Table S2. Visualization of HRP-labeled proteins was performed using enzyme-linked chemifluorescence (ThermoFisher Scientific, Waltham, MA, USA) and quantified using ImageJ software. Data were normalized to control in order to compare different experiments.

### Immunofluorescence

Cells plated onto glass coverslips were incubated with MitoTracker® Deep Red FM (M22426, Molecular Probes®, Life Technologies, Carlsbad, CA, USA) for 45 min and fixed for 10 min with 4% paraformaldehyde (15710-S, Electron Microscopy Sciences). Immunofluorescence staining was performed as previously reported [17]. Antibodies are listed in Supplementary Table S2. Images were acquired in a Zeiss LSM510 confocal microscope using the exact same settings for control and experimental samples and analyzed with ImageJ 1.42q software (NIH, http://rsb.info.nih.gov/ij). Automatic color level correction was used when required to enhance the contrast. Mitochondrial morphology was classified as tubular, mixed, or round (fragmented) according to published criteria [18]. Images were acquired at ×63 magnification, and 1000–5000 cells were counted by two blinded investigators on 16 randomly selected visual fields from at least two independent experiments, using ImageJ. Tyrosine hydroxylase positive neurons were counted over total ßIII-tubulin positive neurons at day 50–70 as previously described [17]. Live images were acquired using the Zoe™ Fluorescent Cell Imager (BioRad, Hercules, CA, USA) at ×20.

### PINK1 Over-expression

Fibroblasts at 70–90% confluence were electroporated with the Neon® Transfection System (Invitrogen™, Waltham, MA, USA), using two pulses of 1500 V for 20 ms, with wild-type *PINK1* (pcDNA-DEST47 PINK1 C-green fluorescent protein (GFP)) [19], from MR Cookson, Addgene no. 13316) or a control GFP plasmid, at 0.5 μg/10^6^ cells. Additional controls in each group received only the pulses. Cells were collected for analysis at 24 and 48 h post-transfection given that expression declined rapidly to baseline levels after 72 h (data not shown). Electroporation in neural cells was done following the same procedure using two pulses of 1000 V. Neurons were collected for analysis at 4 days to minimize the effect of the electroporation on transcriptional changes.

### iPSC and Neuronal Differentiation

Human-iPSC lines from the two Parkinson patients and one carrier and from age-matched control individuals were derived in our laboratory using lentiviral vectors and differentiated as previously described [17] (see Supplementary Fig. S1). The cell lines have been deposited in the Spanish National Cell Bank and are available at http://www.isciii.es/ISCIII/es.

### Data Analysis and Statistics

Data analysis was carried out using GraphPad Prism software (v. 4.0c, La Jolla, CA, USA). One-way or two-way ANOVA with Bonferroni post-hoc tests were used to compare groups. Student’s *t* test was used to detect changes in fold expression whenever data were normalized to control levels. In all experiments using human fibroblast samples, four to five control individuals were assayed together with the two *PINK1* ^*-*exon7/del^ patients and the two *PINK1* ^*-*exon7^ carriers or with the two G309D homozygous and one heterozygous carrier in at least two independent determinations. For neuronal experiments, data from three independent differentiations of the three mutant iPSC lines were included with two to three control pluripotent lines. In addition to the iPSC lines reprogrammed for this study, control cell lines [17] were differentiated and analyzed in parallel. Data in the figures represent the mean ± SEM of two to four independent experiments. The threshold for significance was set at *p* < 0.05.

## Results

### Characterization of Parkinson’s Disease *PINK1* Mutant Fibroblasts

**Fig. 1:**
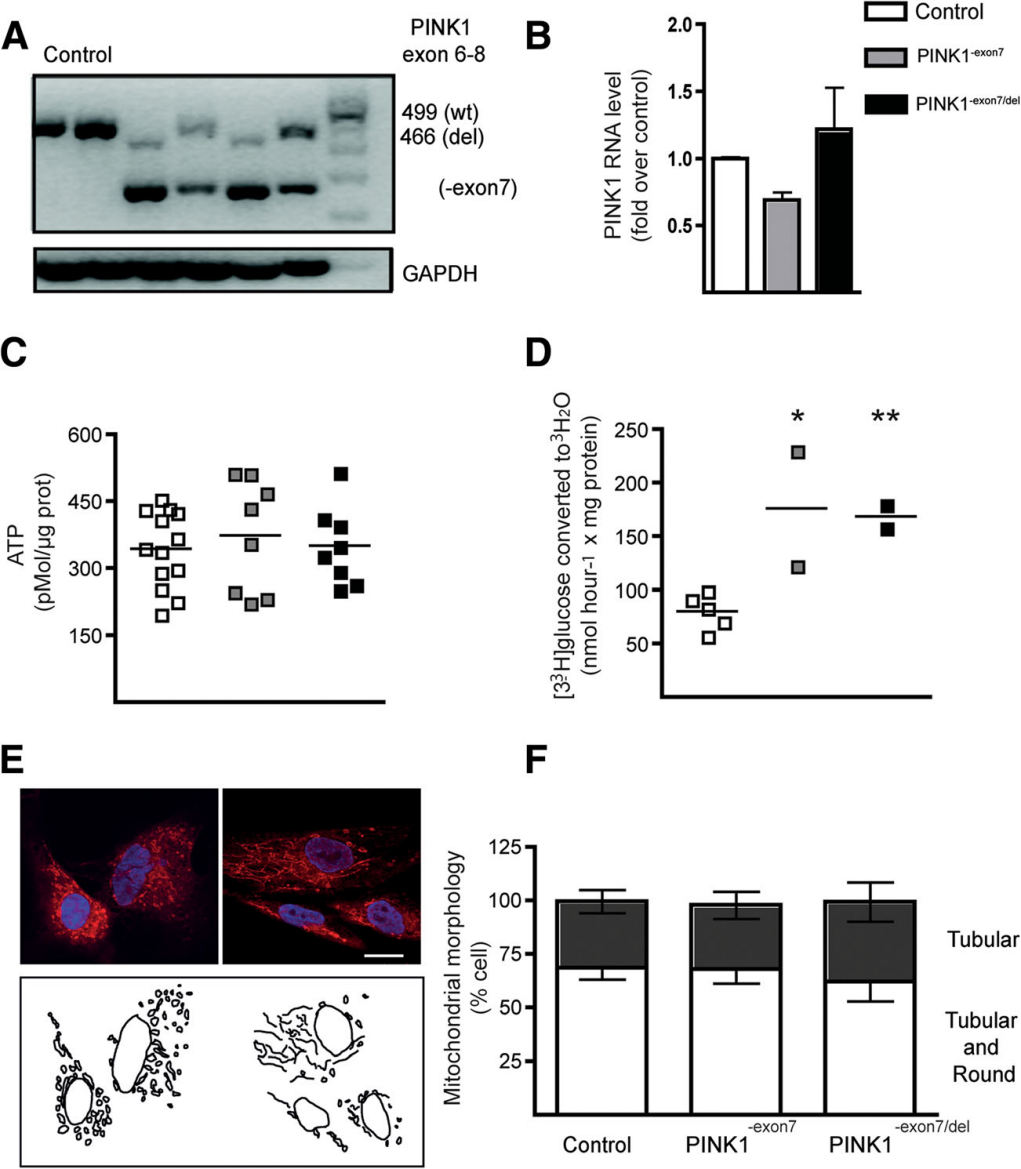
PINK1 mutant fibroblasts characterization. **a** PCR analysis and agarose gel electrophoresis in controls, PD patients (PDP1 and PDP3), and carriers (PDP2 and PDP4) show the exon 7 loss and an additional 33-bp deletion in PINK1^-exon7/del^ samples (PDP1 and PDP3). GAPDH was used as the reference gene. **b** Quantitative RT-PCR determination of *PINK1* RNA levels in control (*N* = 4), carrier (*N* = 2), and patient (*N* = 2) samples. Columns represent the mean ± SEM of three independent experiments in the same samples. **c** ATP levels were not different in control, carrier, and mutant fibroblasts. *Scatter plot graphic* of three independent experiments. **d** Glycolysis flux, measured as the rate of [3-^3^H] glucose incorporation into ^3^H_2_O, in control (*N* = 5), carrier (*N* = 2), and mutant (*N* = 2) fibroblasts, was increased in PINK1 mutant fibroblasts. Experiments were done in triplicate. One-way ANOVA and post-hoc analysis *p < 0.05 and **p < 0.01. **e** Examples of mitochondrial network tubularity visualized with MitoTracker® staining showing a tubular and round network in the *left panel* and a tubular in the *right one*. **f**. Quantification of tubularity in fibroblast samples showed no differences between genotypes. Mitochondrial morphology was assessed in randomly selected fields, and >150 cells were analyzed. *Columns* represent the mean ± SEM of three independent experiments. *Scale bar* 50 μm

We established primary cultures of fibroblasts obtained from dermal biopsies of healthy subjects and individuals carrying modifications in the *PINK1* gene that result in the inactivation of the normal kinase function [14]. Sample verification was confirmed by conventional PCR analysis (Fig. 1a) that identifies the shorter transcripts corresponding to the deletion (del) and the exon 7 skipping (-exon7) in the compound heterozygous patients (PINK1^-exon7/del^, PDP1 and PDP3) and heterozygous carriers (PINK1^-exon7^, PDP2 and PDP4). In spite of the mutations, *PINK1* RNA expression was similar in mutant, carriers, and control fibroblasts (Fig. 1b). Baseline ATP levels were not different between groups (Fig. 1c). Nevertheless, using a sensitive method for glycolytic flux assessment, we detected an increase in the glycolytic rate in *PINK1* mutants and carriers (Fig. 1d), as recently described in mouse *Pink1* KO cells [16]. We next examined the morphology of the mitochondrial network using MitoTracker®; semi-quantitative analysis of tubularity (Fig. 1e, f) showed no differences between genotypes. This does not exclude subtle alterations in mitochondrial morphology or function, but it is in agreement with previous studies in fibroblasts carrying other *PINK1* mutations [20]. We analyzed the expression levels of proteins involved in mitochondrial fusion (MNF2) and fission (DNM1L and MFF) to explore whether the lack of morphological changes in mitochondrial tubularity was due to compensatory adjustments in their levels. The results are shown in Supplementary Fig. S1 and revealed minor changes in fusion/fission dynamics that can reflect compensatory adaptations to the lack of PINK1 kinase function in fibroblasts. These results are consistent with previous studies that have used human fibroblasts to investigate disease and compensatory mechanisms in genetic PD [21].

### Effect of *PINK1* Over-expression in Fibroblasts

**Fig. 2:**
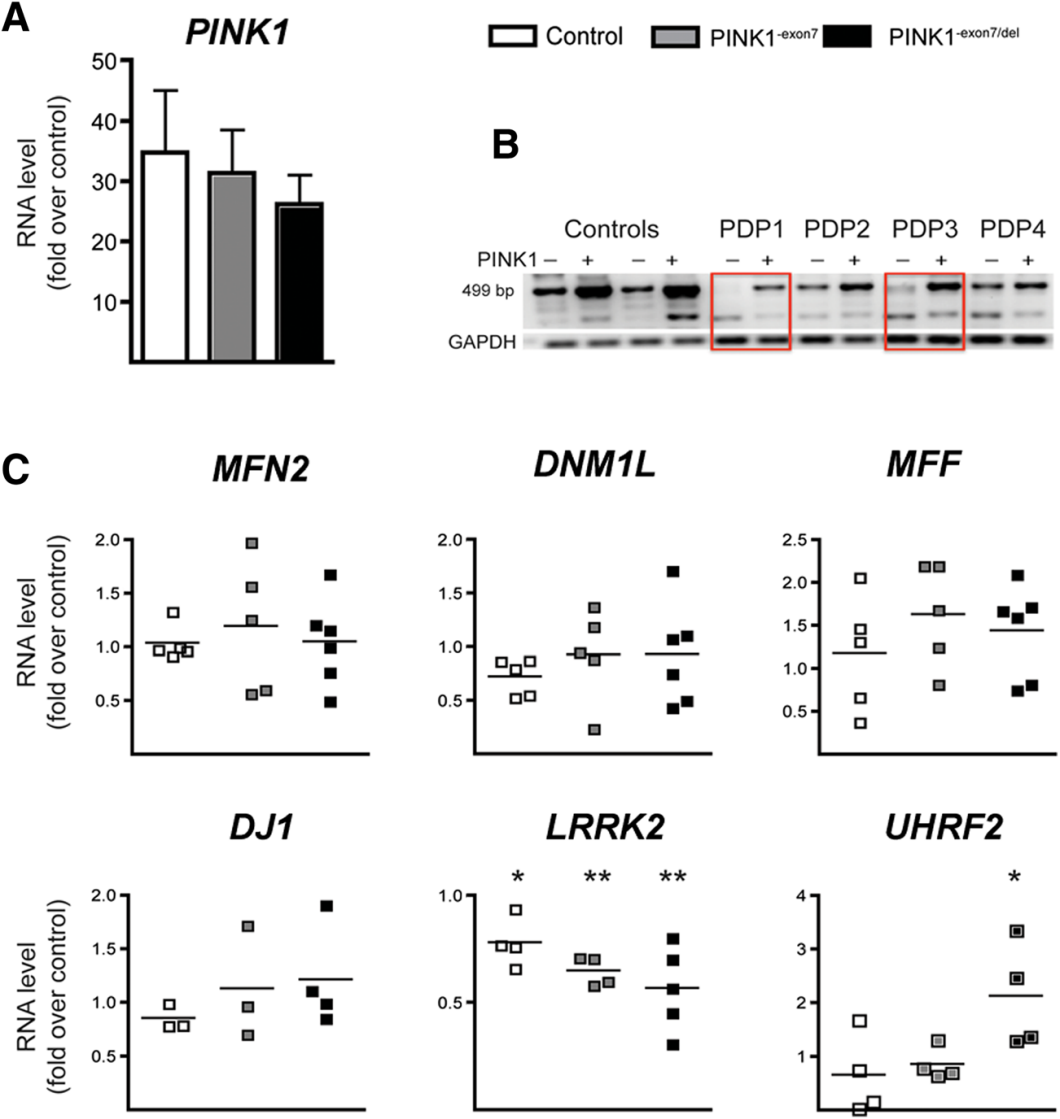
Over-expression of wild-type *PINK1* downregulates *LRRK2*. **a** *PINK1* RNA levels analyzed by quantitative RT-PCR. Average increase by qPCR was not different across groups (31 ± 4.5-fold increase over mock GFP transfection). **b** A representative image of an agarose gel electrophoresis showing the expression of the 499 bp band corresponding to the exons 6 to 8 of PINK1 in the PINK1^-exon7/del^ samples (PDP1 and PDP3; *boxed*) after electroporation with PINK1.GFP. **c** RNA levels analyzed by quantitative RT-PCR in fibroblasts after PINK1 over-expression. All data are expressed as fold change over mock (GFP) transfected samples. *Scatter plot graphics* of two to three independent experiments in control (*N* = 2), carrier (*N* = 2), and mutant (*N* = 2) fibroblasts. One-way ANOVA. *p < 0.05 and **p < 0.01

To identify changes causally related to PINK1 deficiency, we evaluated the capacity of wild-type *PINK1* over-expression to modify the gene expression profile in *PINK1* ^-exon7/del^ mutant fibroblasts. Following electroporation, average *PINK1* levels determined by qPCR were elevated, 31 ± 4.5-fold over control cells, with no differences across genotypes in two independent experiments (Fig. 2a) and a clear expression of a 499 band corresponding to the wild-type *PINK1* exons 6–8 in transfected mutant fibroblasts (boxed in Fig. 2b). Transient transfection of wild-type *PINK1* did not modify the levels of genes involved in mitochondrial dynamics such as mitofusin 2 (*MFN2*), and the pro-fission *DNM1L* or *MFF* genes (Fig. 2c). Likewise, there were no significant changes in *DJ1* (*PARK7*) or *Parkin* (*PARK2*, not shown), whereas, interestingly, we found a significant decrease in *LRRK2* (*PARK8*). In view of these results, we analyzed the expression of *UHRF2*, an E3 ligase reported to be repressed in LRRK2^G2019S^ mutant neurons [22] and found that it was upregulated (Fig. 2d), suggesting that the changes in *LRRK2* expression in this paradigm are relevant.

### LRRK2 Expression in *PINK1* Mutant Fibroblasts

**Fig. 3:**
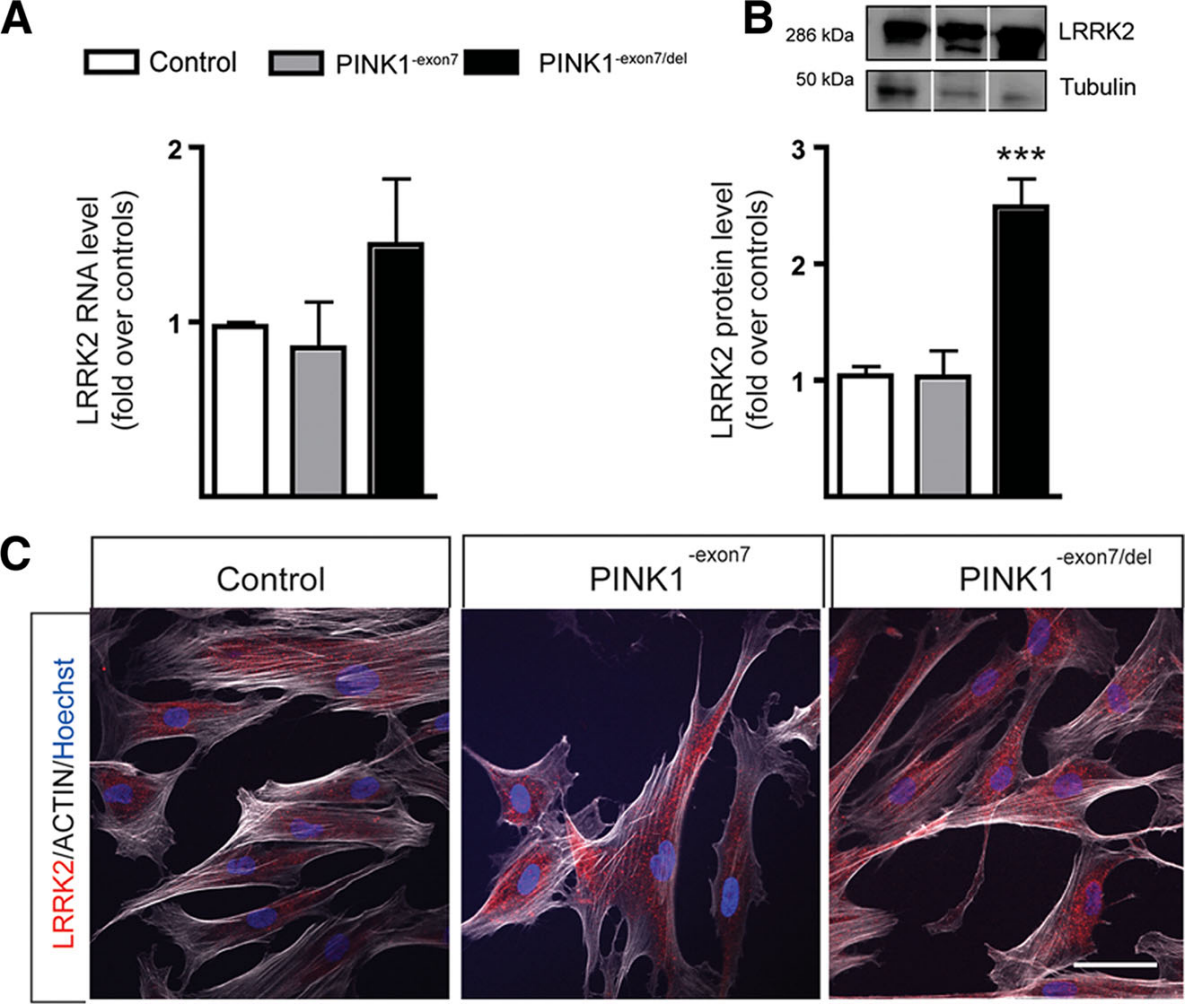
LRRK2 baseline expression in fibroblasts. **a** *LRRK2* RNA levels by qPCR were not significantly different in control (*N* = 4), carrier (*N* = 2), and mutant (*N* = 2) fibroblasts examined in triplicate. **b** Representative blot and quantification of LRRK2 protein level in the same samples showed a significant increase in *PINK1* mutants (2.49 ± 0.24; ***p < 0.001). *Columns* represent the mean ± SEM of four independent experiments. **c** LRRK2 subcellular distribution showed a similar pattern in all genotypes. Cells were counterstained with phalloidin (actin, *white*) to visualize the cytoskeleton. *Scale bar* 50 μm

Since we identified a significant decrease in *LRRK2* in *PINK1* over-expression experiments, we went on to study the baseline expression of LRRK2 in mutant fibroblasts. *LRRK2* RNA was elevated in PINK1^-exon7/del^ fibroblasts although not significantly different from the control (Fig. 3a). Although some transcripts have been shown to increase with aging in fibroblasts and other tissues, we have not found any correlation between age and *LRRK2* mRNA levels in fibroblasts from control individuals studied in our laboratory (age range 17–63 years; *R* ^2^ = 0.082, *p* = 0.42, data not shown). LRRK2 protein was remarkably increased in PINK1^-exon7/del^ samples, with no change in PINK1^-exon7^ fibroblasts (Fig. 3b). LRRK2 sub-cellular distribution was similar in all groups (Fig. 3c).

### LRRK2 Expression in *PINK1* Mutant Neurons

**Fig. 4:**
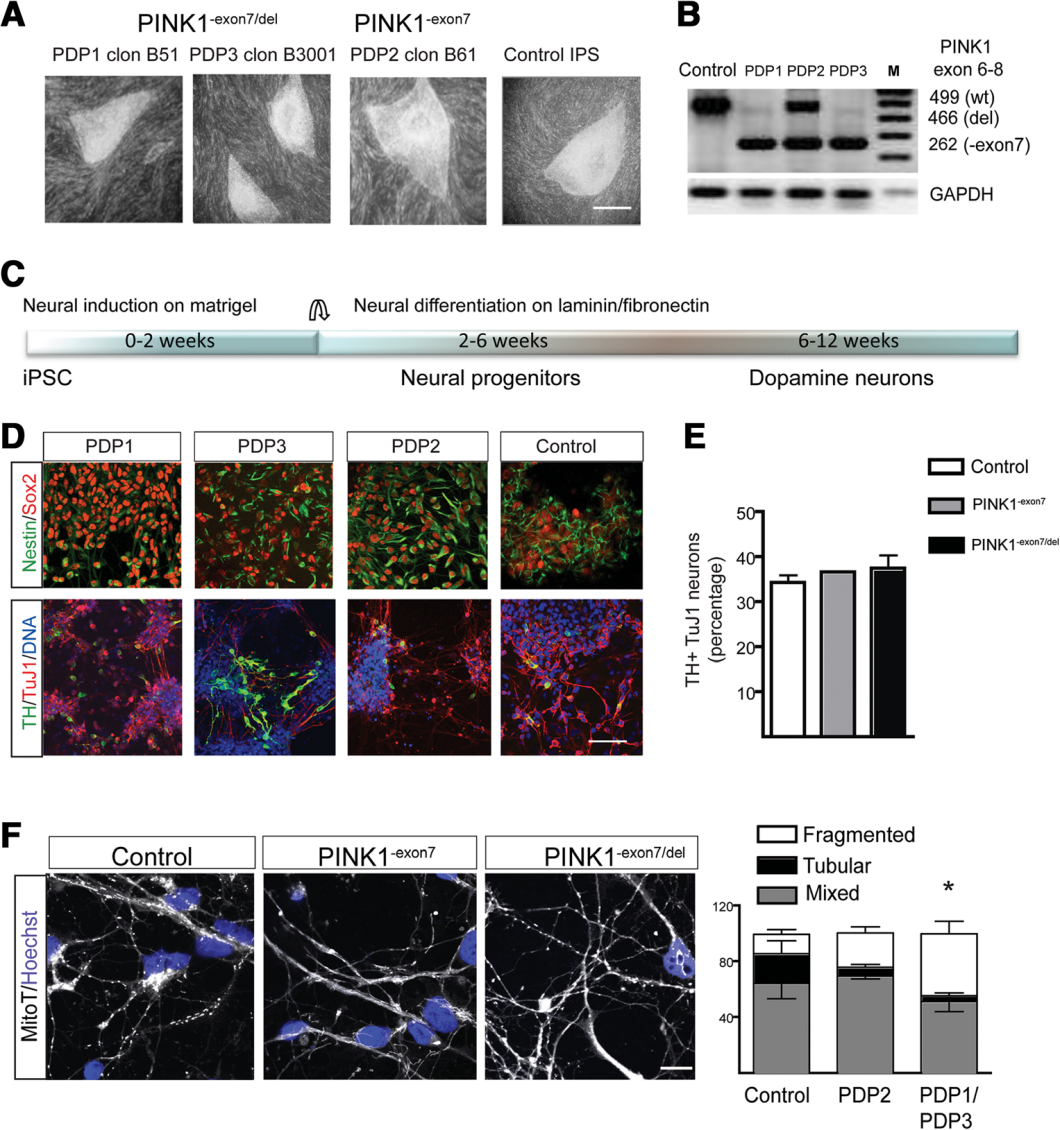
PINK1 iPSC neuron characterization. **a** Representative bright field images of iPSC colonies from selected clones from two Parkinson’s disease patients (PDP1 and PDP3) and one asymptomatic carrier (PDP2) and a control used for differentiation. *Scale bar* 0.5 mm. **b** PCR analysis of PINK1 showed the same splicing pattern in the iPS cell lines as in the original fibroblasts. **c** Schematic representation of the differentiation protocol and the two maturation stages used for analysis. **d** Confocal images show ubiquitous expression of Sox2 (*red*) and Nestin (*green*) at the neural progenitor stage and βIII-tubulin (TuJ1, *red*) and TH (*green*) at the neuronal stage. Scale bar 50 μm. **e** Quantification of TH positive neurons (over TuJ1) at the neuronal stage showed no differences across different cell lines in two independent experiments. **f** Representative images of MitoTracker® labeling in 8-week-old neurons from control (*N* = 2), carrier (*N* = 1), and mutant (*N* = 2) lines. *Scale bar* 10 μm. Quantification of cells presenting predominantly tubular, mixed, or fragmented mitochondrial morphology showed that there were more neurons with fragmented network in the mutant cultures (one-way ANOVA and post-hoc analysis, *p* < 0.05)

Our findings in fibroblasts suggest that *PINK1* and *LRRK2* may act in a convergent pathway, with PINK1 regulating LRRK2 expression. Thus, to identify whether LRRK2 may be a factor contributing to the degeneration of dopamine neurons in PINK1-PD, we made iPSC lines from the two PINK1^-exon7/del^ patients (PDP1 and PDP3 lines) and one carrier (PDP2) (Supplementary Fig. S2 and Fig. 4a, b). Unfortunately, the other carrier line (PDP4) was lost due to technical problems. Pluripotent cells were differentiated towards dopaminergic neurons using an inductive protocol combining developmental signals as described [17] (Fig. 4c). Because significant changes in the expression of genes and levels of proteins occur during neuronal maturation, we analyzed cultures at two time points, corresponding to neural progenitors (2–6 weeks) and neurons (6–12 weeks) (Fig. 4c). There were no apparent defects either in neural induction in the mutant cells, in agreement with published results for iPSC lines carrying homozygous point mutations in the *PINK1* gene [7, 23, 24], or in the generation of dopamine neurons, with >30% of TH positive neurons in all genotypes (Fig. 4d, e). Analysis of the mitochondrial morphology in mature neurons showed more cells with a fragmented mitochondrial network in the PINK1^-exon7/del^ cultures (Fig. 4f). However, RNA expression level of *MNF2*, *DNM1L*, and *MFF* were not significantly different between genotypes (Supplementary Fig. S3).

**Fig. 5:**
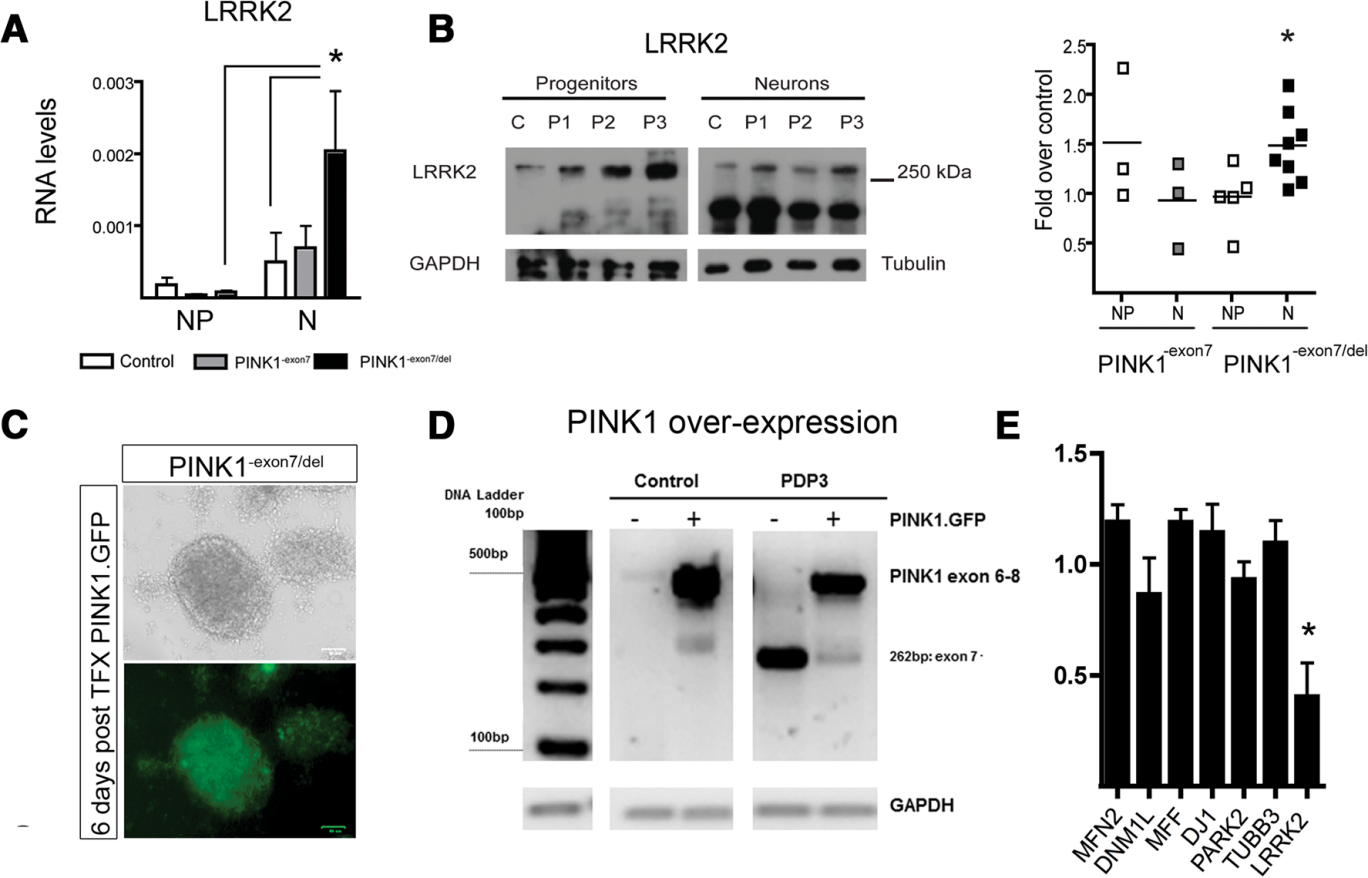
LRRK2 increase in PINK1 iPSC-derived neurons. **a** *LRRK2* RNA levels significantly increased in *PINK1* mutants from the progenitor (*NP*) to the neuronal (*N*) stage (two-way ANOVA, *p* < 0.05) and were higher in mutants than in control neurons (*p* < 0.05) **b** Representative Western blots and corresponding quantification of LRRK2 protein levels at *NP* and *N* stages showed a significant increase in the mutant neurons (*p* < 0.01). **c** Live images of GFP immunofluorescence in *PINK1* mutant NP (in vitro day 35) 6 days after electroporation with wild-type *PINK1.GFP. Scale bar* 60 μm. **d** Representative image of an agarose gel electrophoresis showing the over-expression of the 499 bp band corresponding to the wild-type PCR product (exons 6–8) of PINK1 in electroporated control and PINK1^-exon7/del^ neurons (PDP3). Average PINK1 increase by qPCR was not different across groups. **e** qPCR analysis in three independent over-expression experiments showed a significant downregulation of *LRRK2* (a decrease of 60 ± 9.6% over GFP mock transfection; *p* < 0.05), without inducing any significant changes in fusion/fission genes, *PARK7* and *PARK2*, or βIII-tubulin (*TUBB3*) expression

It is known that *LRRK2* expression is rather low in the developing brain [25]. Indeed, in neural progenitors, *LRRK2* RNA levels were barely detectable and there were no differences between groups. The expression increased at the neuronal stage, but only mutant neurons had significantly higher *LRRK2* levels than progenitors, which were also higher than control neurons (Fig. 5a). At the protein level, the *PINK1* ^-exon7/del^ mutant neurons showed increased protein levels of LRRK2 at the neuronal stage (Fig. 5b), corroborating our findings in fibroblasts in a disease-relevant cell context. Over-expression of *PINK1* wild-type in *PINK1* ^-exon7/del^ mutant cells was performed at the end of neural progenitor stage (5–6 weeks) with good survival and robust expression of the GFP reporter 1 week after transfection (Fig. 5c) that was confirmed by the presence of a strong band corresponding to the full length PCR product of exons 6–8 (Fig. 5d). Like in the fibroblasts, over-expression of wild-type *PINK1* induced a marked decrease in *LRRK2* expression, with no effect on the expression of neuronal βIII-tubulin, *TUBB3*, other *PARK* genes or mitochondrial gene expression (Fig. 5e).

**Fig. 6:**
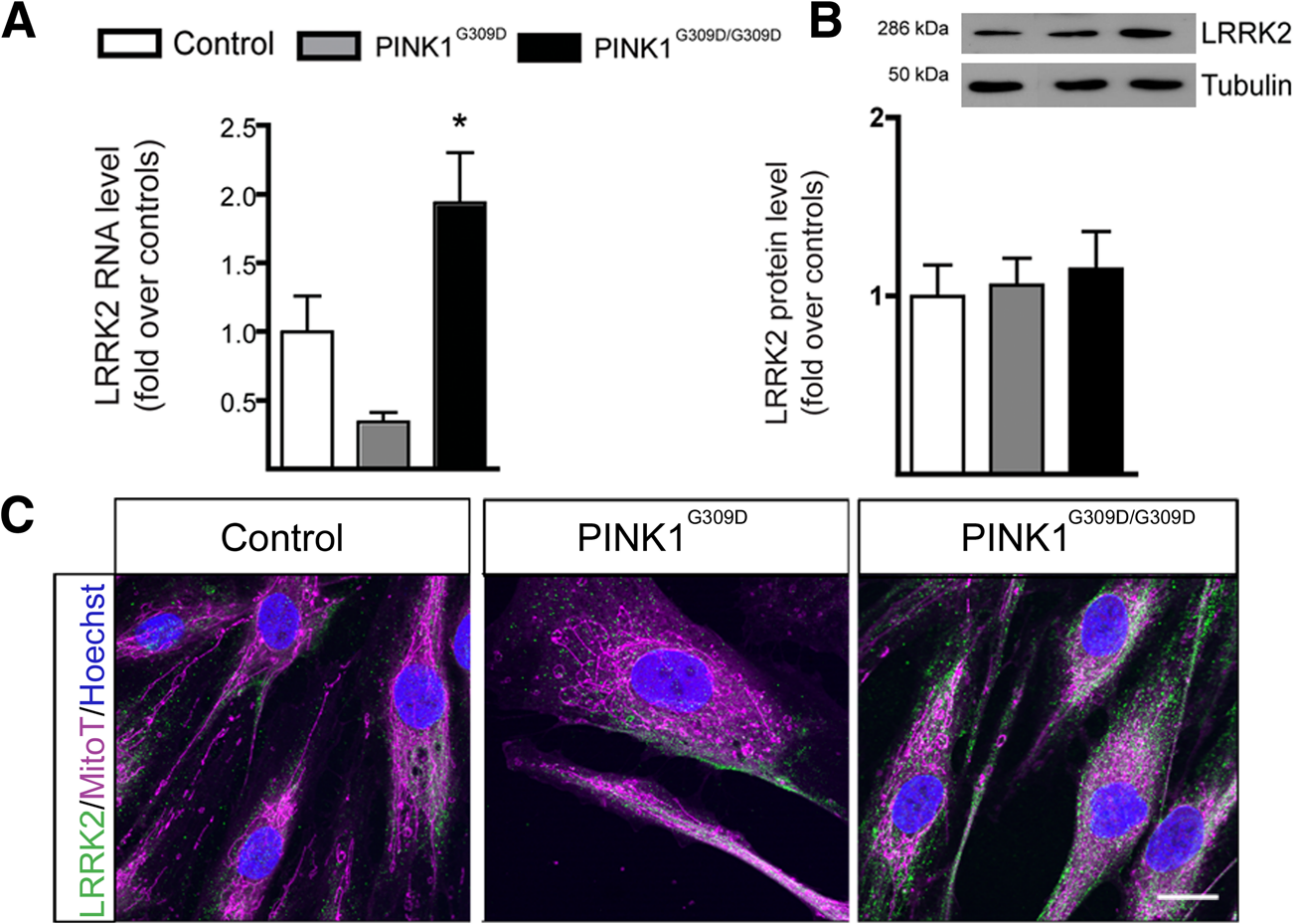
LRRK2 baseline expression in PINK1 G309D fibroblasts. **a** *LRRK2* RNA levels analyzed by quantitative RT-PCR in fibroblasts with PINK1 G309D homozygous mutations (*N* = 2) were significantly higher than in controls (*N* = 5). *Columns* represent the mean ± SEM of three independent experiments. *p < 0.05. **b** LRRK2 protein levels in the same samples were not significantly different from control in this analyses; experiments were done in triplicate and a representative blot is shown above the quantification over the loading control. **c** Representative images of LRRK2 (*green*) and the mitochondrial network labeled with MitoTracker® (*magenta*) in the G309D fibroblasts. *Scale bar* 10 μm

To establish the relevance of LRRK2 deregulation in PINK1 mutants, we next examined LRRK2 levels in human fibroblasts harboring another mutation in *PINK1*, G309D (see Table 1), located in exon 4, that causes a modest decrease in kinase function with no change in protein stability [3, 19, 20]. The glycolytic rate was higher in the homozygous samples (105.7 ± 1, *N* = 2) than in controls (75.4 ± 6.9, *N* = 3, *p* < 0.05, data not shown). Fibroblasts from both PD patients had higher *LRRK2* RNA levels (Fig. 6a), although in these samples, the protein levels were not significantly different from control (Fig. 6b). The mitochondrial network appeared to be normal (Fig. 6c).

## Discussion

In this study, we sought to define a disease-related in vitro phenotype in human cells with PD-associated *PINK1* mutations. We did not find consistent mitochondrial alterations as hypothesized but found instead a remarkable deregulation of LRRK2, revealing a previously under-recognized connection between these two *PARK* genes in human cells. We corroborated this association in fibroblasts from a second family with a different mutation in *PINK1*.

Our data suggest that PINK1 exerts, directly or indirectly, an inhibitory effect on *LRRK2* at the transcriptional level. Indeed, over-expression of wild-type *PINK1* decreased *LRRK2* levels in control and mutant cells. In mutant fibroblasts and neurons, *LRRK2* deregulation resulted in increased LRRK2 protein level.

Abnormal protein synthesis, cytoskeletal dynamics, and mitochondrial transport have all been implicated in LRRK2 pathogenesis [26]. Studies in *C. elegans* [12] and *Drosophila* [27] suggest an antagonistic effect of lrk-1 and pink-1 [13] with clear, if complex, interactions between these two genes, not unlike in our study.

In agreement with previous studies [8, 20, 28], PINK1-patient fibroblasts showed only subtle signs of mitochondrial dysfunction with normal levels of ATP—at the expense of an increased glycolytic rate—and no conspicuous alterations in the mitochondrial network. Similar findings have been documented for other *PARK* genes, like *Parkin* [29] and *LRRK2* [30]. The net effect of PINK1 kinase deficiency on fusion/fission dynamics is a matter of controversy as different model organisms show opposite effects (increased tubularity or increased fission) [10]. It is worth noting that there are remarkable differences between vertebrate (mouse and zebrafish) and invertebrate (fly and worm) models regarding the effects of *PARK* genes on mitochondrial dynamics. Analysis of proteins involved in fusion and fission mitochondrial dynamics in PD fibroblasts showed alterations that are consistent with activation of compensatory mechanisms in the mutants but could also reflect a defective inhibition of fission through TRAP1 [31, 32].

Recent studies have proposed patient fibroblasts as an adequate system to investigate disease mechanisms and compensatory pathways in genetic PD [21], but we took advantage of reprogramming technology to generate iPSC and neurons from these patients and validate our findings in a disease-relevant cell type. iPSC-derived neurons offer the benefit of preserving cell-type specific endogenous expression and transcriptional regulation of the mutated gene. While modeling a complex, age-related and mostly sporadic disorder such as PD is challenging, iPSC harboring monogenic, early-onset variants like PINK1 may provide valuable insights into disease mechanisms [33]. Also critical is the capacity to generate a significant proportion of target cells from the iPSC. In this study, we obtained about 30% of TH+ neurons from all genotypes.

*PINK1* ^-exon7/del^ mutant neurons had a significant increase in the percentage of cells with fragmented mitochondria suggesting a shift in mitochondrial dynamics towards fission. However, this should be interpreted with caution, because in neurons, many different pathways can result in this phenotype. Indeed, we did not find any significant changes in fusion or fission genes, besides a uniform developmental increase in their expression.

Interestingly enough, we observed a remarkable upregulation of LRRK2 in the *PINK1* ^-exon7/del^ mutant neurons. Furthermore, like in fibroblasts, transient over-expression of wild-type *PINK1* effectively downregulated *LRRK2* expression without having any effect on neuronal markers, mitochondrial dynamics, or other *PARK* genes.

LRRK2 has been implicated in a very broad range of cellular pathways, and the precise mechanisms leading to neuronal degeneration in PD-associated *LRRK2* mutations remain to be defined. Nonetheless, increased levels of LRRK2 appear to be directly related to the pathogenicity/toxicity of PD-related mutations, at least for the *LRRK2* ^G2019S^ mutation [34], which is one of the mutations most frequently associated with PD. Importantly, not only mutations but also several polymorphisms in *LRRK2* are associated to an increased risk of PD. Our data suggest that PINK1 and LRRK2 act on a common pathogenic pathway in an antagonistic manner.

Both LRRK2 and PINK1 have been found to modulate the phosphorylation state of several Rab GTP-ases [35, 36], and PD-related mutations could therefore affect vesicle trafficking. In this regard, it is also interesting that over-expression of Rab1 could rescue the *SNCA* mutant phenotype in invertebrate and mammalian models and in human neurons carrying PD-associated *SNCA* triplication [37, 38], indicating that several *PARK* genes with quite distinct clinical manifestations (early onset, typical PD, or dementia with Lewy body) may share common molecular mechanisms [39].

Further downstream, LRRK2 has recently been found to directly phosphorylate p53 (TP53), thus acting in a pro-apoptotic role—phosphorylation of p53 leads to transcriptional activation of pro-apoptotic genes such as *BAX*, *PUMA* (*BBC3*), *NOXA* (*PMAIP1*), and others, as well as activation of transcriptional independent pro-apoptotic mechanisms—in a tissue-specific manner [40]. It has been proposed that either LRRK2 or p53 could initiate cell death in dopamine neurons [41]. PINK1 negatively regulates p53 activity through activation (phosphorylation) of histone deacetylases, which could account for its pro-survival and anti-apoptotic role [42]. Therefore, in *PINK1* mutant neurons, the lack of functional PINK1 could perhaps lead to activation of pro-apoptotic mechanisms through upregulation of LRRK2, although this remains to be proven. A better understanding of LRRK2 function is required to identify novel ways to re-establish this balance in susceptible neurons in PINK1-associated and, perhaps, more broadly in PD.

In conclusion, we report a novel role of PINK1 modulating the levels of LRRK2 in patient fibroblasts and neurons. Although *LRRK2* is one of the genes that is most frequently associated with PD, its role in early-onset recessive forms of the disease had not been previously determined. Our results suggest a convergent pathway for these *PARK* genes, acting in an antagonistic manner, and broaden the involvement of LRRK2 in the pathogenesis of PD.

## Electronic supplementary material

Fig. S1. Analysis of mitochondrial dynamics related genes in fibroblasts. **a**. qPCR of *MFN2*, *DNM1L* and *MFF* showed a decrease in mitofusin2 and an increase in fission factor in the PINK1 mutants. **b**. At the protein level WB analysis of MFN2 showed a small increase, suggesting that the transcriptional decrease might be compensatory to a decreased degradation, while (C) MFF protein expression was elevated. Columns represent the mean ± SEM of four independent experiments in the same samples. One-way ANOVA; * *p* < 0.05 and ** < p 0.001. **d**-**e**. Representative confocal images, showing typical distribution and partial co-localization of D. MFN2 and E. MFF (*green*) with MitoTracker® (magenta) in all fibroblasts. Scale bar: 20 μm (GIF 627 kb)

High Resolution Image (TIFF 53235 kb)

Fig. S2. Characterization of pluripotency of PINK1 mutant iPSC lines. **a**. Fingerprinting analyses of the clones used in the study and their corresponding fibroblasts; BJ: unrelated control. **b**. Karyotypes. **c**. iPSC colonies showed robust expression of pluripotent markers such as OCT4, SSEA-4 and Nanog. **d**. Pluripotency genes were expressed at similar levels than in control human embryonic stem cells (H9). **e**-f. Pluripotency was confirmed in vivo (E) by formation of teratomas in NOD-SCID mice and in vitro (F) by embryoid body formation and tri-lineage differentiation (GIF 214 kb)

High Resolution Image (TIFF 5603 kb)

Fig. S3. Mitochondrial genes expression in PINK1 iPSC-derived neurons. Quantitative PCR analysis of RNA levels of mitochondrial fusion (*MFN2*) and fission (*DNM1L* and *MFF*) genes in neural progenitors (NP) and neurons (N) showed a significant effect of time (developmental stage) and no differences across genotypes. Two-way ANOVA; * *p* < 0.05 and ** < p 0.001 (GIF 27 kb)

High Resolution Image (TIFF 819 kb)

Table S1 (DOCX 22 kb)

Table S2 (DOCX 14 kb)
