# Supplementary figures and images for: LRRK2 Expression Is Deregulated in Fibroblasts and Neurons from Parkinson Patients with Mutations in PINK1
Source: Mol Neurobiol. 2016 Dec 14;55(1):506–16. doi: 10.1007/s12035-016-0303-7 (PMC5808058; doi:10.1007/s12035-016-0303-7)

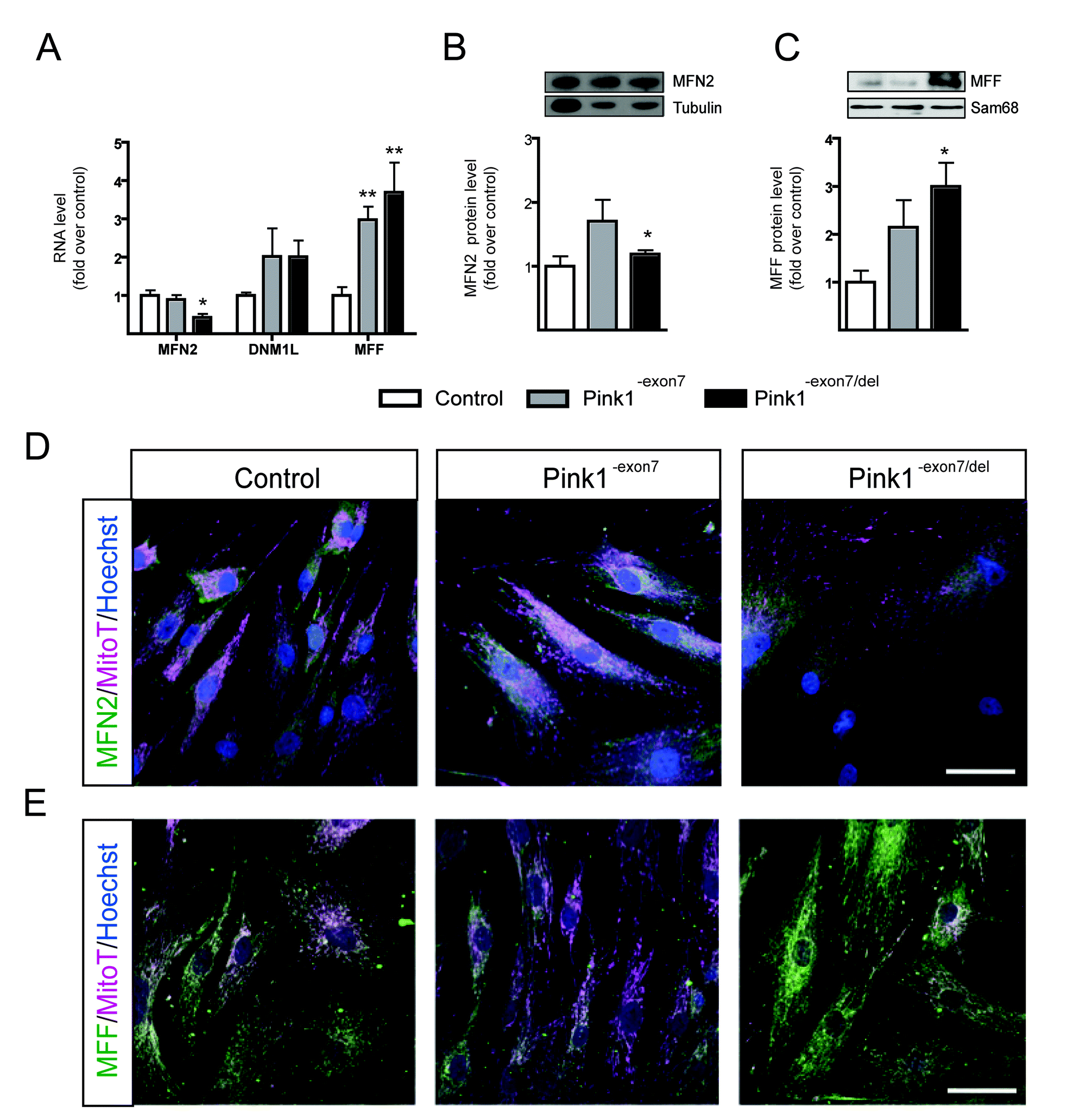

Supplement: Supplementary file 1 — Analysis of mitochondrial dynamics related genes in fibroblasts. a. qPCR of MFN2, DNM1L and MFF showed a decrease in mitofusin2 and an increase in fission factor in the PINK1 mutants. b. At the protein level WB analysis of MFN2 showed a small increase, suggesting that the transcriptional decrease might be compensatory to a decreased degradation, while (C) MFF protein expression was elevated. Columns represent the mean ± SEM of four independent experiments in the same samples. One-way ANOVA; * p < 0.05 and ** < p 0.001. d-e. Representative confocal images, showing typical distribution and partial co-localization of D. MFN2 and E. MFF (green) with MitoTracker® (magenta) in all fibroblasts. Scale bar: 20 μm (GIF 627 kb) [file 12035_2016_303_Fig7_ESM.gif]

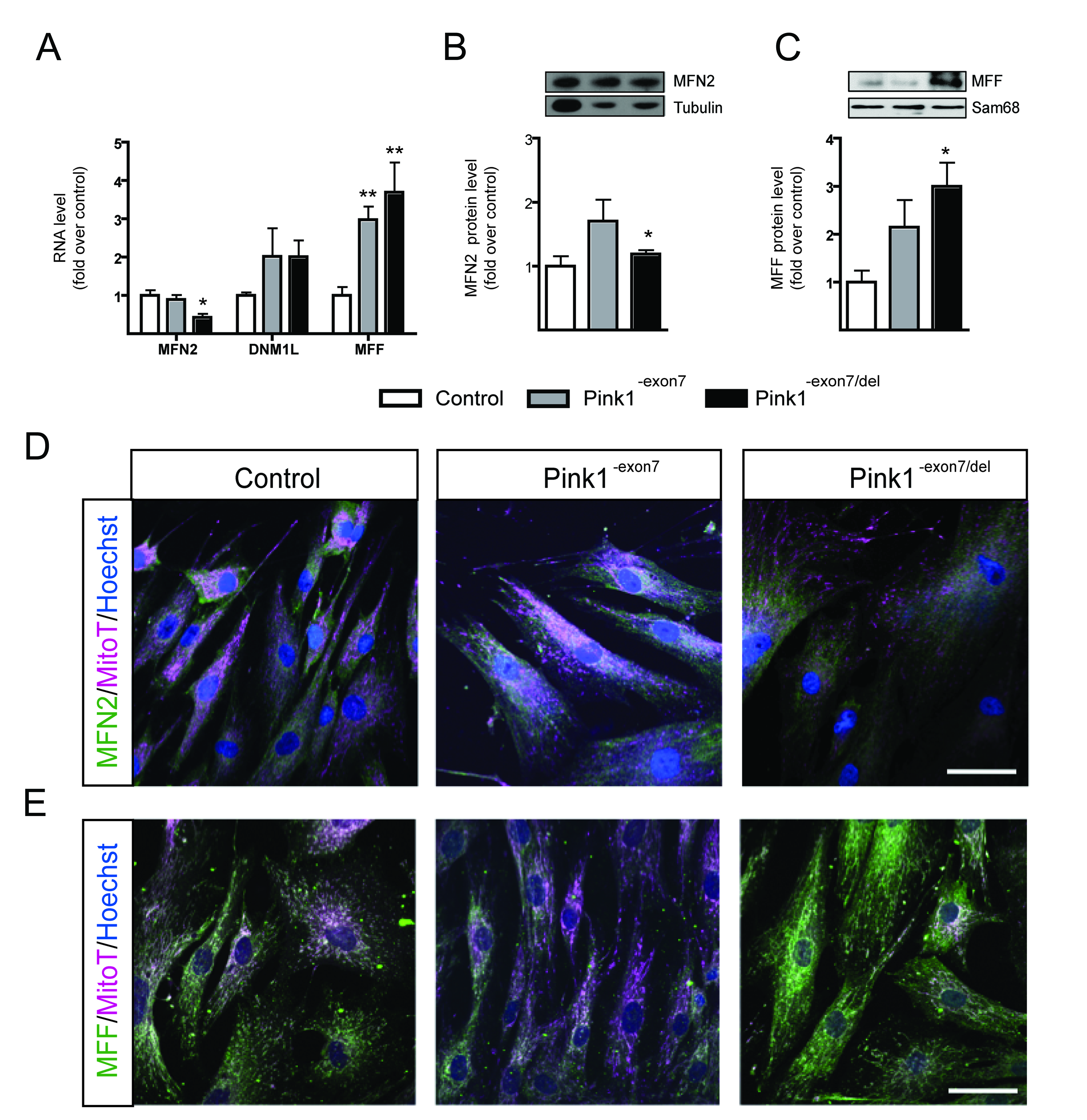

Supplement: Supplementary file 2 — High Resolution Image (TIFF 53235 kb) [file 12035_2016_303_MOESM1_ESM.tif]

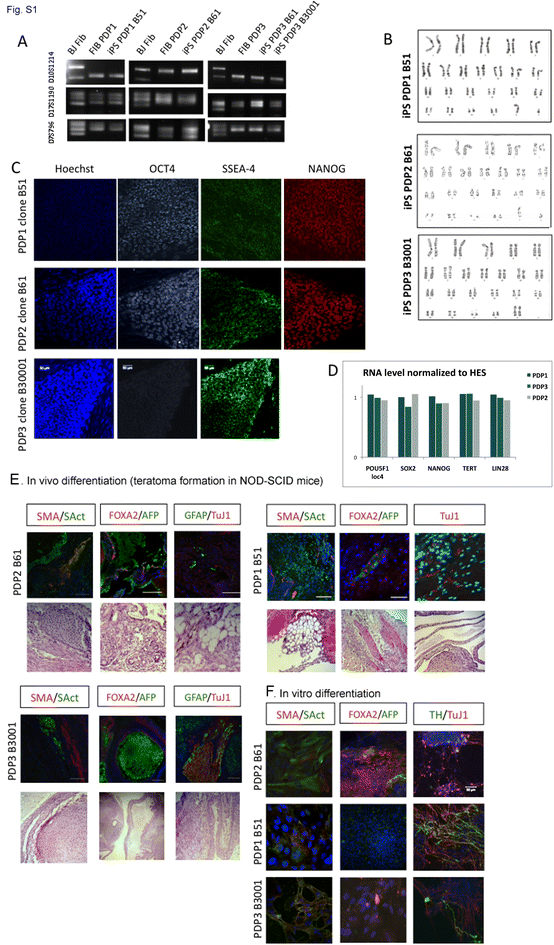

Supplement: Supplementary file 3 — Characterization of pluripotency of PINK1 mutant iPSC lines. a. Fingerprinting analyses of the clones used in the study and their corresponding fibroblasts; BJ: unrelated control. b. Karyotypes. c. iPSC colonies showed robust expression of pluripotent markers such as OCT4, SSEA-4 and Nanog. d. Pluripotency genes were expressed at similar levels than in control human embryonic stem cells (H9). e-f. Pluripotency was confirmed in vivo (E) by formation of teratomas in NOD-SCID mice and in vitro (F) by embryoid body formation and tri-lineage differentiation (GIF 214 kb) [file 12035_2016_303_Fig8_ESM.gif]

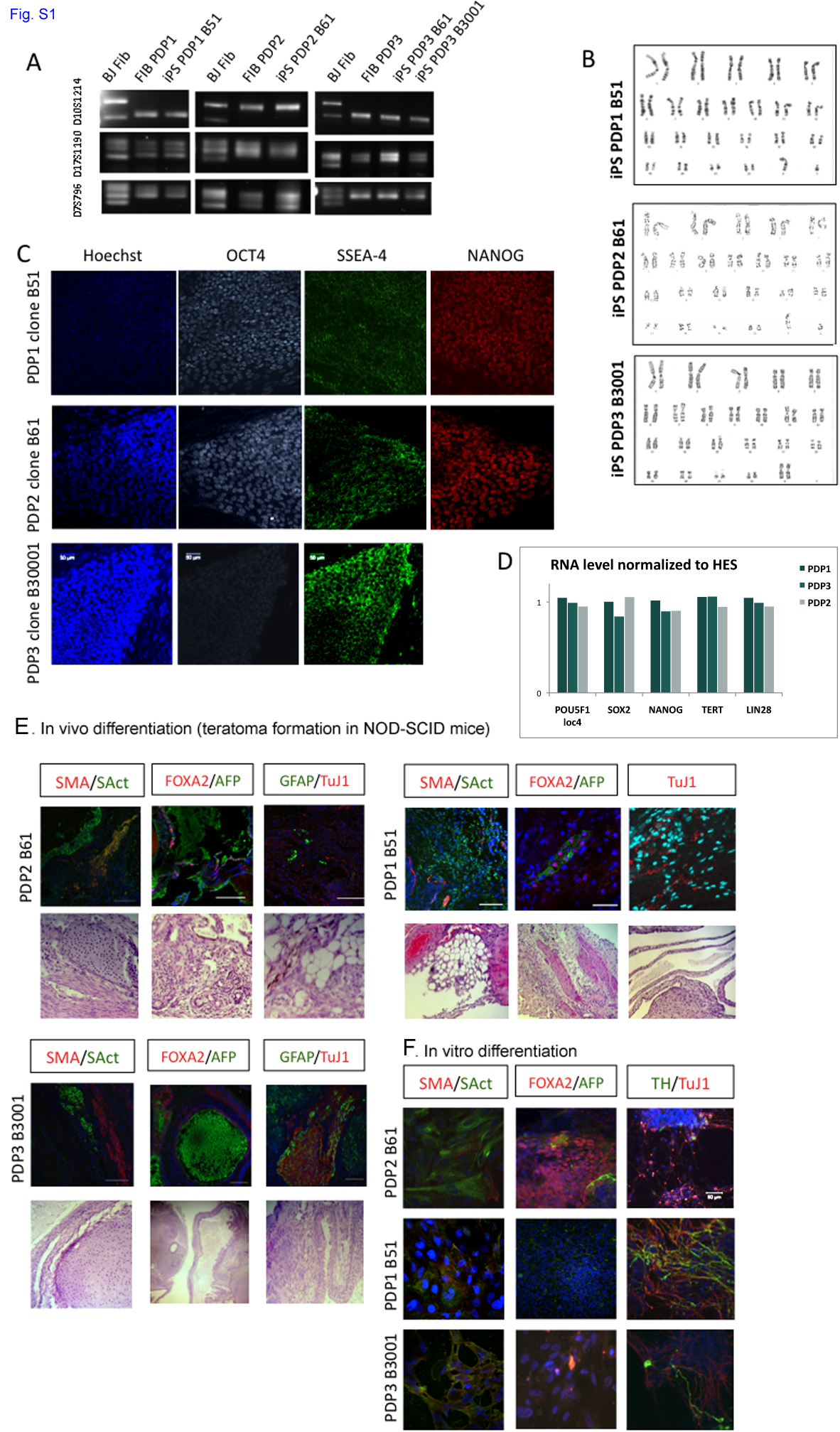

Supplement: Supplementary file 4 — High Resolution Image (TIFF 5603 kb) [file 12035_2016_303_MOESM2_ESM.tif]

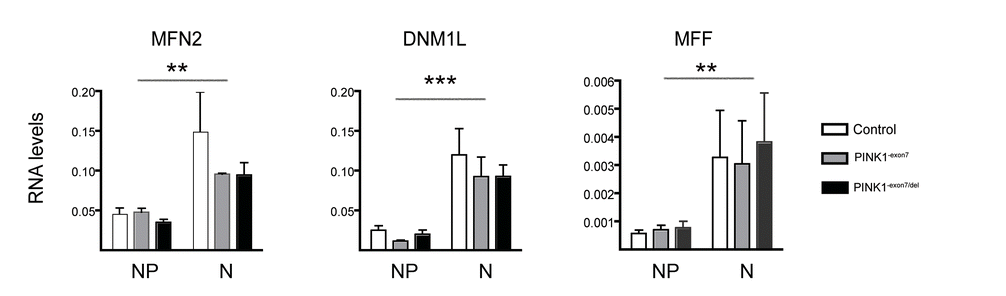

Supplement: Supplementary file 5 — Mitochondrial genes expression in PINK1 iPSC-derived neurons. Quantitative PCR analysis of RNA levels of mitochondrial fusion (MFN2) and fission (DNM1L and MFF) genes in neural progenitors (NP) and neurons (N) showed a significant effect of time (developmental stage) and no differences across genotypes. Two-way ANOVA; * p < 0.05 and ** < p 0.001 (GIF 27 kb) [file 12035_2016_303_Fig9_ESM.gif]

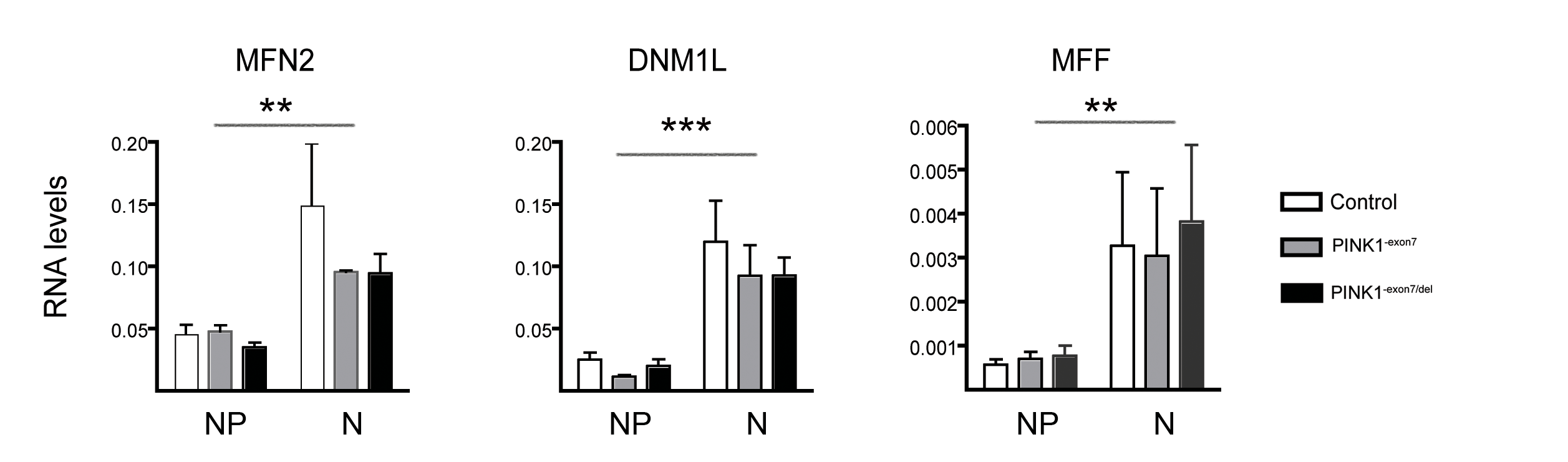

Supplement: Supplementary file 6 — High Resolution Image (TIFF 819 kb) [file 12035_2016_303_MOESM3_ESM.tif]
