# Supplementary material for: LRRK2 Expression Is Deregulated in Fibroblasts and Neurons from Parkinson Patients with Mutations in PINK1
Source: Mol Neurobiol. 2016 Dec 14;55(1):506–16. doi: 10.1007/s12035-016-0303-7 (PMC5808058; doi:10.1007/s12035-016-0303-7)
Supplement: Supplementary file 7 — (DOCX 22 kb) [file 12035_2016_303_MOESM4_ESM.docx]

***Supplementary Table 1.* List of primers used in the study**

| **Symbol** | **Description** | **Forward primer** | **Reverse primer** |
| --- | --- | --- | --- |
| ***PINK1*** | Phosphatase and tensin homologue (Pten)-induced Kinase 1 | GCTTGGGACCTCTCTTGGAT | CGAAGCCATCTTGAACACAA |
| ***PINK1*** | PINK1  exons 6-8 | ATCGCAGATTTTGGCTGCT | ACTTCTCTGTGAGCCTGTTGG |
| ***MFN2*** | Mitofusin 2 | GACCCCGTTACCACAGAAGA | GCAGAACTTTGTCCCAGAGC |
| ***MFF*** | Mitochondrial fission factor | TGAAGCACAGAAGCAGATAGTGC | GGTGTACATAACAACACGGACACTGC |
| ***DNM1L*** | Dynamin 1-like (DRP1) | GCAGGAACTCGGCATATTCT | GCTCGAGAATTTCGTAGGCA |
| ***UHRF2*** | Ubiquitin-Like With PHD And Ring Finger Domains 2 | TAAAGAAAGCTCCGAGGGTAGG | CTGGGTGACAGAGCCAGAC |
| *LRRK2* | Leucine repeat rich Kinase 2 (PARK8) | TCCAGATCAACCAAGGCTCACCAT | AGGCTGCTCGGTAAACTGATCCAA |
| *PARK7* | DJ-1 | GTCATTTGTCCTGATGCCAGC | TCAGATAAATTCTGTGCGCCC |
| *PARK2* | Parkin | CTGCGACACCACCAACAG | TGGATTGCACTTGAATCTGTG |
| *SNCA* | Alpha-synuclein | TCCAGAATTCCTTCCTGTGG | GAAGACAGTGGAGGGAGCAG |
| *TUBB3* | Beta3 tubulin | AGTCGCCCACGTAGTTGC | CGCCCAGTATGAGGGAGAT |
| *GAPDH* | Glyceraldehyde-3-phosphate dehydrogenase | TGCACCACCAACTGCTTAGC | GGCATGGACTGTGGTCATGAG |

Primers for PCR and qPCR were taken from the NIH database (<http://primerdepot.nci.nih.gov/>) or from previous publications [1-3]. Guideline for symbols and description of human genes can be found at GeneCards (<http://www.genecards.org/>).

1. Aguila JC, Blak A, van Arensbergen J, Sousa A, Vazquez N, Aduriz A, Gayosso M, Lopez Mato MP, Lopez de Maturana R, Hedlund E *et al*: **Selection Based on FOXA2 Expression Is Not Sufficient to Enrich for Dopamine Neurons From Human Pluripotent Stem Cells**. *Stem Cells Transl Med* 2014, **3**(9):1032-1042.

2. Lopez de Maturana R, Aguila JC, Sousa A, Vazquez N, Del Rio P, Aiastui A, Gorostidi A, Lopez de Munain A, Sanchez-Pernaute R: **Leucine-rich repeat kinase 2 modulates cyclooxygenase 2 and the inflammatory response in idiopathic and genetic Parkinson's disease**. *Neurobiology of aging* 2014, **35**(5):1116-1124.

3. Samaranch L, Lorenzo-Betancor O, Arbelo JM, Ferrer I, Lorenzo E, Irigoyen J, Pastor MA, Marrero C, Isla C, Herrera-Henriquez J *et al*: **PINK1-linked parkinsonism is associated with Lewy body pathology**. *Brain* 2010, **133**(Pt 4):1128-1142.
