# Supplementary material for: LRRK2 Expression Is Deregulated in Fibroblasts and Neurons from Parkinson Patients with Mutations in PINK1
Source: Mol Neurobiol. 2016 Dec 14;55(1):506–16. doi: 10.1007/s12035-016-0303-7 (PMC5808058; doi:10.1007/s12035-016-0303-7)
Supplement: Supplementary file 8 — (DOCX 14 kb) [file 12035_2016_303_MOESM5_ESM.docx]

***Supplementary Table 2*. List of antibodies used in the study**

| **Antibody** | **Type** | **Dilution** | **Reference** |
| --- | --- | --- | --- |
| MFN2 | Rabbit Polyclonal | 1:200 IF  1:1000 WB | M6319, Sigma |
| MFF | Rabbit Polyclonal | 1:500 IF  1:1000 WB | 17090-1-AP, Proteintech Europe |
| LRRK2 | Rabbit Polyclonal | 1:500 IF  1:1000 WB | Novus Biologicals  NB-300-58771 |
| LRRK2 | Rabbit Monoclonal | 1:1000 WB | 3516-1 (c8L-8 MJFF) Epitomics |
| Alpha-Synuclein | Mouse Monoclonal | 1:1000 WB | MA1-12874 Thermo Scientific |
| Sam68 | Rabbit Polyclonal | 1:1000 WB | sc-333 Santa Cruz Biotech |
| a-Tubulin | Mouse Monoclonal | 1:1000 WB | 3873S, Cell Signaling |
| β-actin | HRP conjugated | 1:1000 WB | ab499000, Abcam |
| GAPDH | HRP conjugated | 1:1000 WB | sc-20357HRP, Santa Cruz Biotech. |
| MitoTracker® Deep Red FM |  | 1:5000 IF | M22426 Molecular Probes |
| Phalloidin® | Alexa 568 conjugated | 1:1000 IF | A12380, Molecular Probes |
| Tyrosine Hydroxylase | Sheep Polyclonal | 1:1000 IF | Pel-Freez Biologicals |
| β- III Tubulin (TUJ1) | Mouse Monoclonal | 1:1000 IF | mms-435p, Covance |
| Sox2 | Goat Polyclonal | 1:500 IF | GT15098 Neuromics |
| Nestin | Mouse Monoclonal | 1:250 IF | MO15012 Neuromics |
| Alexa-fluor conjugated secondary antibodies | Donkey anti rabbit/mouse/sheep/goat | 1:500 IF | Molecular Probes |
